# Treatment of very elderly glioblastoma patients ≥ 75 years of age: whom to treat

**DOI:** 10.1007/s11060-023-04518-w

**Published:** 2023-11-30

**Authors:** Peter Baumgarten, Georg Prange, Marcel A. Kamp, Daniel Monden, Vanessa Neef, Franziska Schwarzer, Daniel Dubinski, Nazife Dinc, Katharina J. Weber, Markus Czabanka, Elke Hattingen, Michael W. Ronellenfitsch, Joachim P. Steinbach, Christian Senft

**Affiliations:** 1 Department of Neurosurgery, University Hospital Frankfurt, Goethe University, Frankfurt, Germany; 2 Department of Neurosurgery, University Hospital Jena, Friedrich Schiller University, Jena, Germany; 3 Department of Anesthesiology, Intensive Care Medicine and Pain Therapy, University Hospital Frankfurt, Goethe University, Frankfurt, Germany; 4 Neurological Institute (Edinger Institute), University Hospital Frankfurt, Goethe University, Frankfurt, Germany; 5 Department of Neuroradiology, University Hospital Frankfurt, Goethe University, Frankfurt, Germany; 6 Department of Neuro-Oncology, University Hospital Frankfurt – Goethe-University, Frankfurt, Germany; 7 German Cancer Consortium (DKTK), partner site Frankfurt/Mainz, Frankfurt, Germany; 8 German Cancer Research Center (DKFZ), Heidelberg, Germany; 9 Frankfurt Cancer Institute (FCI), Goethe University, Frankfurt, Germany; 10 University Cancer Center (UCT), Goethe University Hospital, Frankfurt, Germany; 11 Present Address: Department of Neurosurgery, University Hospital Jena, Friedrich Schiller University, Am Klinikum 1, D-07747 Jena, Germany; 12 Present Address: Centre for Palliative and Neuro-palliative Care, Brandenburg Medical School Theodor Fontane and Faculty of Health Sciences Brandenburg, Campus Rüdersdorf, Rüdersdorf bei Berlin, Germany; 13 Present Address: Department of Neurosurgery, Hannover Medical School, Hannover, Germany; 14 Present Address: Department of Neurosurgery, University Medicine Rostock, Rostock, Germany

**Keywords:** Elderly, Glioblastoma, Glioma, Very elderly

## Abstract

**Purpose:**

The prognosis of patients ≥ 75 years suffering from glioblastoma is poor. Novel therapies are usually reserved for patients ≤ 70 years. In an aging population, treatment of very elderly patients remains a challenge.

**Methods:**

Between 2010 and 2018, a total of 977 glioblastoma patients were treated at our institution. Of these, 143 patients were ≥ 75 years at diagnosis. Primary procedure was surgical resection or biopsy followed by adjuvant treatment, whenever possible. We retrospectively investigated overall survival (OS) and potential prognostic factors influencing survival, including Karnofsky Performance Status (KPS), surgical therapy, adjuvant therapy as well as MGMT promotor status.

**Results:**

In very elderly patients, median age was 79 years (range: 75–110). Biopsy only was performed in 104 patients; resection was performed in 39 patients. Median OS for the entire cohort was 5.9 months. Univariate analysis showed that KPS at presentation (≥ 70 vs. ≤60), surgery vs. biopsy, adjuvant chemotherapy and adjuvant radiotherapy were significantly associated with OS (6 vs. 3, p < 0.0111; 12 vs. 4, p = 0.0011; 11 vs. 4, p = 0.0003 and 10 vs. 1.5 months, p < 0.0001, respectively). Multivariate analysis confirmed adjuvant radiotherapy (p < 0.0001) and chemotherapy (p = 0.0002) as independent factors influencing OS.

**Conclusion:**

For very elderly patients, the natural course of disease without treatment is devastating. These patients benefit from multimodal treatment including adjuvant radiotherapy and chemotherapy. A beneficial effect of resection has not been demonstrated. Treatment options and outcomes should be thoughtfully discussed before treatment decisions are made.

**Supplementary Information:**

The online version contains supplementary material available at 10.1007/s11060-023-04518-w.

## Introduction

Glioblastoma is the most frequent malignant brain tumor with a poor prognosis even in younger patients [1]. Common treatment strategies include neurosurgical tumor resection followed by concomitant and adjuvant radiotherapy either with temozolomide according to the European Organization for Research and Treatment of Cancer (EORTC)-22,981/26,981 / National Cancer Institute of Canada (NCIC) CE3 trial [1, 2]. Although patients > 70 years of age were excluded in this pivotal trial, benefit of therapy is conveyed in elderly patients as well: the prospective NOA-08, Nordic and the CCTG CE.6/EORTC 26,062 − 22,061 phase III trials addressed less aggressive treatment protocols in an elderly glioblastoma patient population [3–5]. Among other things, these studies established the value of O6-methylguanine-DNA methyltransferase (*MGMT*) promoter methylation status as a prognostic and therapeutic marker. Recent guidelines, such as the European Association for Neuro-Oncology (EANO) guideline, recommended to treat IDH-wildtype glioblastoma patients ≥ 70 years with surgery followed by hypofractionated radiotherapy for MGMT-promoter-unmethylated tumours and followed by the standard concomitant and adjuvant radiotherapy with temozolomide or temozolomide alone [6]. However, optimal treatment of elderly glioblastoma patients is still a matter of debate.

Aim of the present retrospective study was to establish outcome data in a non-selected cohort of elderly patients and to identify prognostic factors for this patient subgroup.

## Materials and methods

### Patients

We retrospectively analyzed 977 patients treated surgically for the former diagnosis “glioblastoma, WHO grade IV”, in our hospital, between 01/2010 and 12/2018. Surgical treatment was defined as either resection, biopsy or biopsy followed by resection. Treatment for recurrent tumor was reported as well. Histological diagnosis was confirmed by at least two board certified neuropathologists following standard HE stains and immunohistochemical staining for glial fiber acidic protein (GFAP) and Ki67 antigen, according to the then current WHO classification [1]. Methylation specific polymerase chain reaction (mPCR) for the MGMT promoter was performed in all primary cases following routine protocols. MGMT-promoter methylation was either stated as “methylated”, “not methylated” or “not conclusive” if mPCR did not show conclusive results with good positive and negative control in two runs with patient material. Patient data was retrieved from the electronical patient reports.

### Outcome parameters

All MRI images were obtained by contrast-enhanced 1.5T or 3T MRI. For detection of tumor and tumor progression, non-contrast-enhanced and contrast-enhanced T1, T2-weighted, diffusion weighted imaging and fluid attenuated inversion recovery sequences were evaluated. Both, a senior neurosurgeon and neuroradiologist independently evaluated the MR scans. A tumour in-brain progression was diagnosed according to the Response Assessment in Neuro-Oncology (RANO) criteria [7].

All patients were routinely followed in regular 3-month intervals or shorter, if recommended by our institutional multidisciplinary neurooncology tumor board. General clinical performance was assessed according to Karnofsky Performance Status (KPS) [8, 9]. Treatment decisions were made according to patients’ preferences following tumor board recommendation.

Overall survival was defined as time span from confirmation of the histopathological diagnosis (i.e. date of biopsy or tumor resection) until death due to any cause.

### Statistical analyses

For statistical analyses, patients younger than 75 years of age were excluded. Statistical analysis and figure editing were performed using JMP 14.0 software (SAS, Cary, NC, USA), GraphPad Prism 6 (GraphPad Software Inc., La Jolla, USA) and Gimp2. Survival analyses were performed using Kaplan-Meier analyses. For evaluation of KPS the patient cohort was dichotomized between good and poor status with a cutoff at ≥ 70%. For Kaplan-Meier survival analyses we used Wilcoxon and Log-rank test. For multivariate analyses a proportional hazard ratio was calculated. To compare ratios in different groups, likelihood-ratio and Pearson Chi² were applied. To compare KPS in different treatment groups, MannWhitney U test was used for non-parametric testing. Significance level alpha was set as p ≤ 0.05 for all tests.

### Ethics

The study was approved by the ethics committee of the university hospital of Frankfurt and the University Cancer Center (UCT) Frankfurt/Main (EC number 4/09, project ethical vote SNO_15_2019).

## Results

### Patient population

Of a total of 977 patients, 143 patients were 75 years or older at primary surgery and thus defined as very elderly. In very elderly patients, median age was 79 years (range: 75–110). The data describing the cohort in the primary situation is summarized in Table 1. Sex, MGMT promoter methylation status and the admission of chemotherapy was equally distributed in the resection and biopsy only group. Preoperative KPS ≥ 70% and the probability to receive adjuvant irradiation were significantly higher in the resection group (Table 1).

**Table 1:** Summary of patient characteristics

	Biopsy only	Resection	Total	*p*-Value
	104 (72.7%)	39 (27.3%)	143	log-rank / Pearson
**Gender**			143	0.8369 / 0.8368
- male	58 (55.8%)	21 (53.8%)	79 (55.2%)	
- female	46 (44.2%)	18 (46.2%)	64 (44.8%)	
**KPS preopative**			143	0.0021 / 0.0026 **
- ≥ 70	48 (46.2%)	29 (74.4%)	77 (53.8%)	
- < 70	56 (53.8%)	10 (25.6%)	66 (46.2%)	
**MGMT**			109	0.5423 / 0.5423
- methylated	28 (38.4%)	16 (44.4%)	44 (40.4%)	
- unmethylated	45 (61.6%)	20 (55.6%)	65 (59.6%)	
- inconclusive	31	3	34	
**Chemotherapy**			118	0.1244 / 0.1226
- yes	30 (36.1%)	18 (51.4%)	48 (40.7%)	
- no	53 (63.9%)	17 (48.6%)	70 (59.3%)	
**Irradiation**			116	0.0073 / 0.0098 **
- yes	47 (58.0%)	29 (82.9%)	76 (65.5%)	
- no	34 (42.0%)	6 (17.1%)	40 (34.5%)	

### Influence of resection, adjuvant chemotherapy, irradiation and preoperative Karnofsky performance scale score (KPS) on overall survival (OS)

**Fig. 1:**
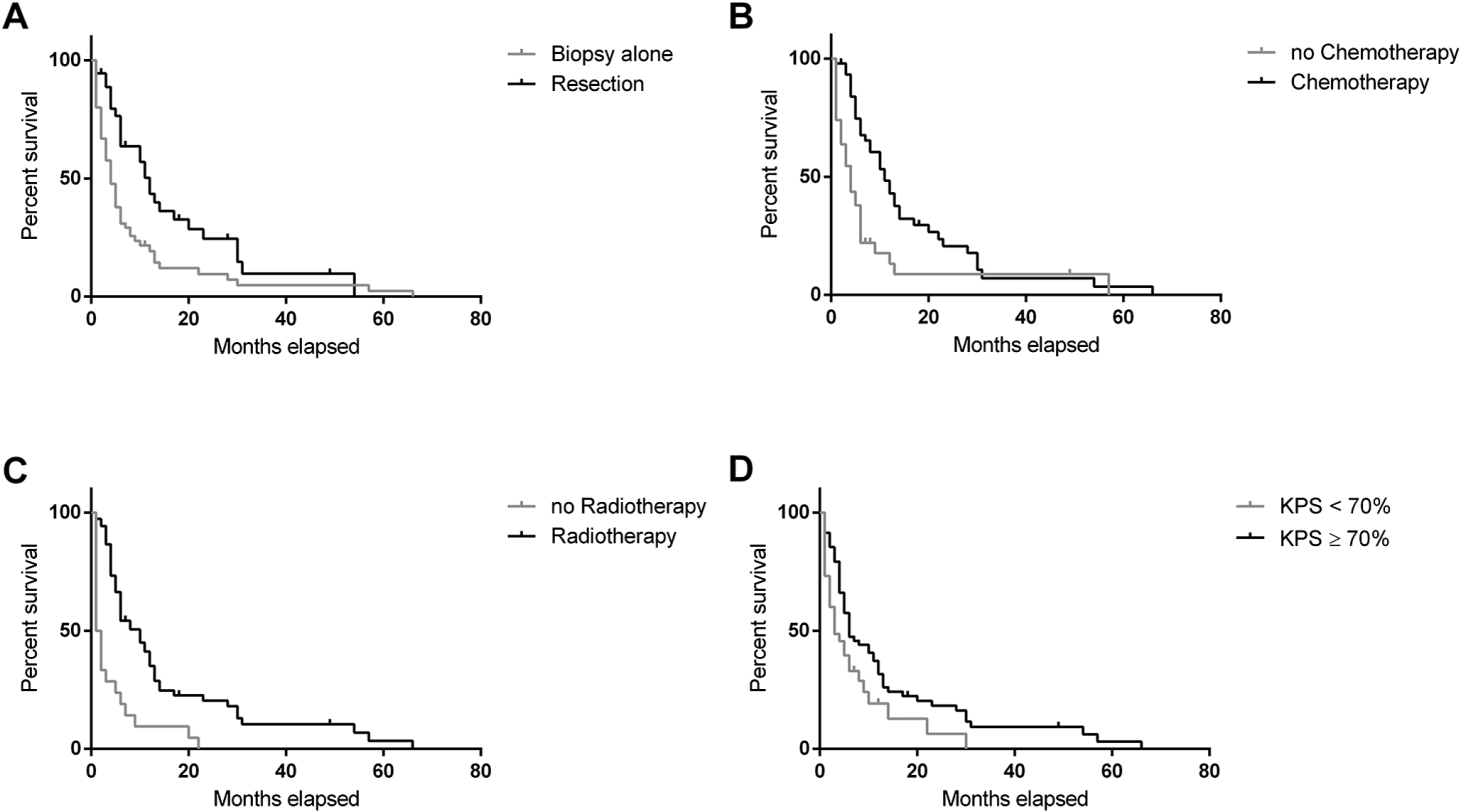
Comparison of overall survival (OS) (**A**) Biopsy alone vs. tumor resection (**B**) No chemotherapy vs. chemotherapy (**C**) No radiotherapy vs. radiotherapy (**D**) Preoperative KPS ≥ 70 vs. < 70

Comparing patients who underwent resection or resection after biopsy in the primary situation (n = 39) with patients without resection but biopsy only (n = 104), patients undergoing microsurgical resection showed significantly longer OS than patients receiving biopsy only (median survival 12 vs. 4 months, Wilcoxon p = 0.0002, Log Rank p = 0.0011; Fig. 1A). Patients who received adjuvant chemotherapy after resection or biopsy showed significantly longer OS than patients without (n = 48 vs. 70; median survival 11 vs. 4 months, Wilcoxon p < 0,0001, Log Rank p = 0.0003; Fig. 1B). In the same context, adjuvant irradiation after resection or biopsy was associated with significantly longer OS (n = 76 vs. 40; median survival 10 vs. 1.5 months, Wilcoxon p < 0,0001, Log Rank p < 0.0001; Fig. 1C). Preoperative KPS was dichotomized at ≥ 70% / <70%. Patients in the group with higher preoperative KPS showed a significantly higher OS in our cohort (n = 77 vs. 66; median survival 6 vs. 3 months, Wilcoxon p < 0,0036, Log Rank p < 0.0111; Fig. 1D).

### Multivariate survival analyses

In multivariate analyses we compared the effects of surgical treatment, chemotherapy, irradiation and preoperative KPS (Table 2). Independent factors influencing OS were adjuvant irradiation (p < 0.0001) and adjuvant chemotherapy (p = 0.0002) but not preoperative KPS or surgical treatment.

**Table 2:** Multivariate analyses regarding the effects of surgical treatment, adjuvant chemotherapy, adjuvant irradiation and preoperative KPS on OS

	No. Parameters	Likelyhood Ratio Chi²	Probability > Chi²
Adjuvant Irradiation	1	21.3233135	< 0.0001
Adjuvant Chemotherapy	1	14.022966	0.0002
Preoperative KPS ≥ 70%	1	0.93978041	0.3323
Resection vs. Biopsy	1	2.25067413	0.1336

### Distribution of Karnofsky Performance Status (KPS) in the different treatment groups

**Fig. 2:**
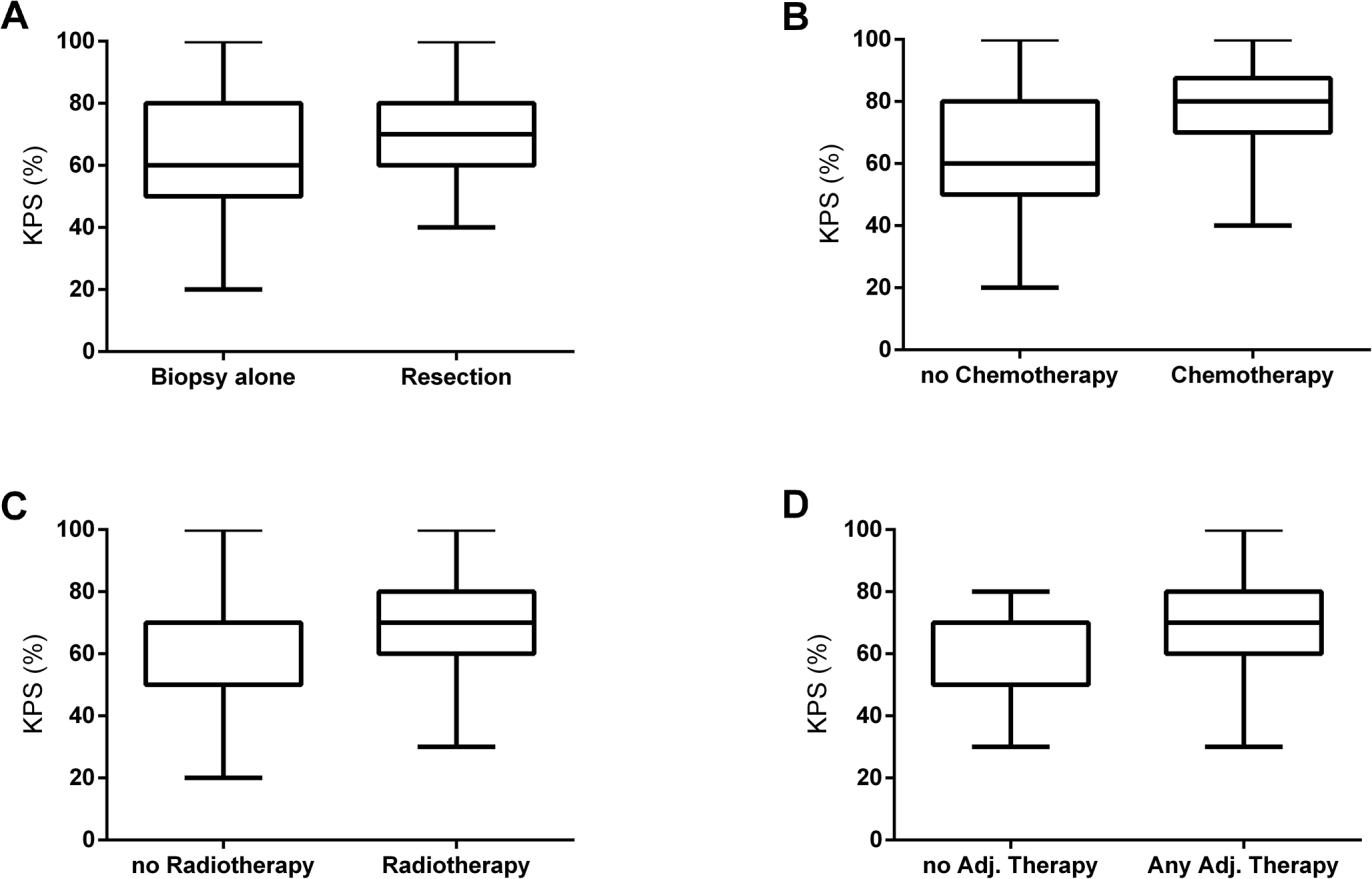
Comparison of Karnofsky Performance Status (KPS) (**A**) Biopsy alone vs. tumor resection (**B**) No chemotherapy vs. chemotherapy (**C**) No radiotherapy vs. radiotherapy (**D**) No adjuvant therapy vs. any adjuvant therapy

KPS was significantly higher in more aggressive treatment regimens: Patients with microsurgical resection showed higher KPS than those with biopsy only (Mann-Whitney U test, p = 0.0045, Fig. 2A), same as patients with adjuvant chemotherapy (Mann Whitney test, p = 0.0001, Fig. 2B) or adjuvant irradiation (Mann-Whitney U test, p < 0.0001, Fig. 2C). Higher KPS was further associated with any adjuvant therapy as compared to no adjuvant therapy (Mann-Whitney U test, p < 0.0001, Fig. 2D).

## Discussion

The present study represents one of the largest series of non-selected very elderly glioblastoma patients reported so far. We identified adjuvant irradiation and chemotherapy as favorable independent factors for the overall survival in very elderly patients.

Although glioblastoma is common in elderly patients, standard therapy has long been defined only for patients under 65 or under 70 years of age. Additionally, the age cut-off for several studies on therapy for glioblastoma in the elderly is set at 65 years [3, 5, 10–13]. Different prospective and randomized trials documented the benefit of complete surgical resection if at the same time new perioperative deficits can be avoided [14–17]. Based on the EORTC-22,981/26,981 / NCIC CE3 trail, standard adjuvant therapy is an adjuvant and concomitant radio-/chemotherapy with temozolomide [2]. For MGMT promoter methylated glioblastomas, intensification of temozolomide chemotherapy by additional administration of CCNU might be beneficial [18]. Interestingly, there were no relevant differences in the distribution of MGMT promoter methylation status in our elderly cohort compared to other studies with a younger patient cohort [3–5]. Based on the (EORTC)-22,981/26,981 / National Cancer Institute of Canada (NCIC) CE3 study, it was assumed that standard therapy is too aggressive for elderly patients, and less aggressive adjuvant therapy concepts were addressed in prospective studies [19].

The NOA-08 trial compared temozolomide chemotherapy alone (100 mg/m^2^ temozolomide, one week on / one week off protocol) with radiotherapy alone (30 × 1.8-2.0 Gy ad 60 Gy) in glioblastoma patients with a KPS > 50 and an age > 65 years. The study established the MGMT promoter methylation as therapeutic marker as temozolomide chemotherapy was associated with a longer event-free survival in MGMT promoter methylated glioblastoma as compared to radiation therapy alone. In contrast, in MGMT promoter unmethylated glioblastoma, radiation was beneficial [3]. The Nordic trial compared temozolomide chemotherapy alone (200 mg/m^2^; 5/28 day cycle), radiation therapy (30 × 2 Gy ad 60 Gy) and hypofractionated radiotherapy (10 × 3.4 Gy ad 34 Gy) in elderly glioblastoma patients ≥ 60 years. The study confirmed the role of the MGMT as therapeutic marker as the overall survival was not inferior in the temozolomide chemotherapy group compared to standard radiation therapy [5]. The CCTG CE.6/EORTC 26,062 − 22,061 phase III trial compared hypofractionated radiotherapy (40 Gy/15 fractions) alone with hypofractionated radiotherapy with concomitant and adjuvant temozolomide in newly diagnosed glioblastoma patients aged ≥ 65 years [4].

In our present analysis Kaplan-Meier analyses suggested that aggressive neurosurgical treatment with resection instead of biopsy alone was associated with favorable outcome referred to overall survival (OS). In contrast, our multivariate analysis showed no benefit in terms of the overall survival for a surgical resection. Again, although prospective and randomized trials have proven the benefit of a complete surgical resection if at the same time new perioperative deficits can be avoided [14–17], such an association has yet not been shown for elderly and geriatric patients. With reference to the ANOCEF trial from 2022, no advantage was demonstrated for tumor resection compared to biopsy alone in patients ≥ 70 years of age in terms of OS. However, there was a significantly better quality of life and PFS for patients following tumor resection [20]. Here, multimorbidity and geriatric limitations could certainly be risk factors for successful surgery. The benefit of a complete resection may not outweigh the increased risk from multimorbidity and geriatric limitations. However, with our data this remains speculative since co-morbidities weren’t systematically recorded. Further prospective and randomized studies assessing the role of surgical and novel adjuvant treatments in geriatric glioblastoma patients are needed.

Independent of the prospective studies on adjuvant therapy in elderly glioblastoma patients mentioned above, the impact of the adjuvant therapy has been analyzed in numerous retrospective studies [21–31]. Elderly patients in good pre- and postoperative clinical condition may have comparable survival rates as younger patients when treated according to standard of care [32]. However, a more aggressive therapy likely results in a higher frequency of side-effects and clinical deterioration. The predictive role of MGMT promoter methylation is well documented in various studies and should therefore be considered [32, 33].

In our cohort, the natural course of the disease proved to be devastating, especially when biopsy without adjuvant treatment was performed. A more aggressive therapy resulted in a better outcome as defined by a longer overall survival, albeit this also being short. However, it should be considered in this context that aggressive treatments can only be offered to patients with a good general condition, guided by the KPS. Our results may help clinicians to counsel their patients regarding the pros and cons of surgery and/or adjuvant therapy.

### Limitations

We acknowledge several limitations of our analysis: (1) All data are derived from a retrospective, single center study. (2) A standard geriatric assessment was neither established in the present study nor in recent prospective randomized trials [3]. Like the general condition as assessed by the KPS, however, geriatric burden most likely has an impact on the ability to cope with surgical and adjuvant therapy, on the quality of life and on the prognosis. Therefore, a high symptom burden in geriatric screening could be a valuable marker for therapy decisions. (3) In the present series, we analysed a homogenous cohort with elderly IDH-wildtype glioblastoma patients and aimed to minimize potential confounders by different brain tumours diagnoses. As a potential confounder, the patient cohort was heterogenous in respect to pre-therapeutic clinical performance and therapeutic protocols, making estimates regarding their effect on overall survival difficult. (4) Tumors were histopathologically classified by the 2016 version of WHO classification. Recently, a new version of the WHO classification was published. However, the differences between versions of the WHO classification can be regarded as rather small with regard to IDH wild-type glioblastomas. Moreover, new and more aggressive therapies of glioblastomas have been introduced, e.g. the adjuvant and concomitant temozolomide and CCNU chemotherapy for patients with MGMT promoter methylated glioblastoma [18] as well as the tumour-treating fields [34]. The impact of the therapies in the treatment of especially elderly patients remains yet unclear. (5) In the present analysis, we did not analyse the side effects, such as hemato-toxicity or treatment effects on neurocognition. (6) Moreover, we did not perform any assessment of quality of life, psycho-oncological and social burden on patients and their relatives. We cannot exclude that the identified prognostic markers might significantly impair patient’s quality of life. This issue might be addressed in further studies.

## Conclusion

The overall outcome of very elderly patients with glioblastoma is limited. In the present study, we identified adjuvant irradiation and chemotherapy as favorable independent factors on overall survival in very elderly patients age ≥ 75 years. In contrast, surgical resection was not associated with improved survival in multivariable analysis. Future studies should analyze the impact of novel adjuvant treatments as well as the impact of geriatric burden and impairment on overall survival and quality of life in elderly glioblastoma patients.

### Electronic supplementary material

Below is the link to the electronic supplementary material.

Supplementary Material 1

## Data Availability

The datasets generated during and/or analysed during the current study are available from the corresponding author on reasonable request.
