# Supplementary material for: Treatment of very elderly glioblastoma patients ≥ 75 years of age: whom to treat
Source: J Neurooncol. 2023 Nov 30;165(3):509–15. doi: 10.1007/s11060-023-04518-w (PMC10752837; doi:10.1007/s11060-023-04518-w)
Supplement: Supplementary file 1 — Supplementary Material 1 [file 11060_2023_4518_MOESM1_ESM.docx]

|  | **Biopsy only** | **Resection** | **Total** |
| --- | --- | --- | --- |
| **Right hemisphere** | 42 (61.8%) | 26 (38.2%) | 68 |
| **Left hemisphere** | 54 (80.6%) | 13 (19.4%) | 67 |
| **Bihemispheric** | 5 (100.0%) | 0 (0.0%) | 5 |

Suppl. Table 1: Distribution of tumor side

|  | **Biopsy only** | **Resection** | **Total** |
| --- | --- | --- | --- |
| **Corpus callosum** | 3 (100.0%) | 0 (0.0%) | 3 |
| **Frontal** | 19 (79.2%) | 5 (20.8%) | 24 |
| **Fronto-insular** | 1 (100.0%) | 0 (0.0%) | 1 |
| **Fronto-parietal** | 4 (80.0%) | 1 (20.0%) | 5 |
| **Fronto-temporal** | 1 (50.0%) | 1 (50.0%) | 2 |
| **Mesencephal** | 1 (100.0%) | 0 (0.0%) | 1 |
| **Multifocal** | 8 (100.0%) | 0 (0.0%) | 8 |
| **Occipital** | 4 (66.7%) | 2 (33.3%) | 6 |
| **Parietal** | 11 (57.9%) | 8 (42.1%) | 19 |
| **Parieto-occipital** | 6 (75.0%) | 2 (25.0%) | 8 |
| **Parieto-temporo-occipital** | 1 (100.0%) | 0 (0.0%) | 1 |
| **Periventricular** | 1 (100.0%) | 0 (0.0%) | 1 |
| **Ponto-mesencephal** | 1 (100.0%) | 0 (0.0%) | 1 |
| **Postcentral** | 2 (100.0%) | 0 (0.0%) | 2 |
| **Precentral** | 3 (100.0%) | 0 (0.0%) | 3 |
| **Temporal** | 25 (67.6%) | 12 (32.4%) | 37 |
| **Temporo-frontal** | 1 (100.0%) | 0 (0.0%) | 1 |
| **Temporo-medial** | 0 (0.0%) | 1 (100.0%) | 1 |
| **Temporo-occipital** | 2 (50.0%) | 2 (50.0%) | 4 |
| **Temporo-parietal** | 6 (60.0%) | 4 (40.0%) | 10 |
| **Thalamic** | 1 (100.0%) | 0 (0.0%) | 1 |
| **Trigonal** | 2 (100.0%) | 0 (0.0%) | 2 |
| **Central** | 0 (0.0%) | 1 (100.0%) | 1 |

Suppl. Table 2: Distribution of tumor location

| **Dosage in Gy** | **Biopsy only** | **Resection** | **Total** |
| --- | --- | --- | --- |
| **2.64** | 1 (100.0%) | 0 (0.0%) | 1 |
| **8.01** | 1 (100.0%) | 0 (0.0%) | 1 |
| **10.68** | 1 (100.0%) | 0 (0.0%) | 1 |
| **16.02** | 1 (100.0%) | 0 (0.0%) | 1 |
| **18.69** | 1 (100.0%) | 0 (0.0%) | 1 |
| **27.63** | 1 (100.0%) | 0 (0.0%) | 1 |
| **34.71** | 1 (100.0%) | 0 (0.0%) | 1 |
| **36** | 1 (100.0%) | 0 (0.0%) | 1 |
| **37.24** | 1 (100.0%) | 0 (0.0%) | 1 |
| **39.6** | 0 (0.0%) | 1 (100.0%) | 1 |
| **39.9** | 5 (71.4%) | 2 (28.6%) | 7 |
| **40** | 0 (0.0%) | 2 (100.0%) | 2 |
| **40.01** | 1 (100.0%) | 0 (0.0%) | 1 |
| **40.05** | 22 (59.5%) | 15 (40.5%) | 37 |
| **42.56** | 0 (0.0%) | 1 (100.0%) | 1 |
| **45** | 1 (100.0%) | 0 (0.0%) | 1 |
| **52** | 1 (100.0%) | 0 (0.0%) | 1 |
| **60** | 5 (50.0%) | 5 (50.0%) | 10 |

Suppl. Table 3: Distribution of radiotherapy protocols
